# Magnetotrichography: Measuring the dc magnetic field produced by hair follicles

**DOI:** 10.1038/s41598-019-52110-y

**Published:** 2019-10-30

**Authors:** Sheraz Khan, David Cohen

**Affiliations:** 1 Radiology, Massachusetts General Hospital, Harvard Medical School, Boston, MA USA; 2 Athinoula A. Martinos Center for Biomedical Imaging, Boston, MA USA; 3 Francis Bitter Magnet Lab, Massachusetts Institute of Technology, Cambridge, MA USA

**Keywords:** Magnetoencephalography, Chemical physics

## Abstract

We here describe the dc magnetic field over the human head produced by healthy hair follicles when the scalp is lightly pressed. This phenomenon was briefly reported decades earlier, where a double-planar SQUID (Superconducting Quantum Interference Devices) gradiometer at a single location was used. We here perform a larger study, using the dcMEG containing 102 double-planar gradiometers covering the whole scalp. The field is displayed as an on-line arrow map over the head, showing the approximate flow of direct current (dc) in the scalp. Standard sets of five arrow maps per subject were measured, where the subject successively pressed parts of their head against the inside of the helmet. These maps were made for 15 normal subjects (5 females), and 2 with alopecia (non-functioning follicles). The directions of “pressed” generating arrows always followed the natural tilt of the follicles, verifying the follicles as generators, with a time constant of about one second. The maximum generator dipole strength was about 24 µA-cm. Scalp electric potentials corresponding to the magnetic signals were masked by much larger electrodermal potentials. Therefore, this magnetic method, called magnetotrichography, is unique in measuring this follicular electrical activity, with possible applications in studying baldness and hair diseases.

## Introduction

The existence of weak fluctuating magnetic fields produced by the human body is well known. For example, the fluctuating magnetic field produced by the human heart (the magnetocardiogram)^1^ and that produced by the human brain (the magnetoencephalogram or MEG)^2^ have been well studied. The steady (dc) magnetic field of the body has also been reported but was less studied. One dc report was a mapping of the dc field over the entire normal body^3^; other reports were of dc from the ischemic heart^4^ and various states of the brain^5–7^. Of the various studies of dc fields of the body, the mapping over the body^3^ showed a unique new phenomenon, which is in the head: when the scalp was lightly pressed on a region containing healthy hair follicles, an external dc magnetic field appeared over that region. The pressing stimulated the follicles to produce a dc, which then generated a dc magnetic field. We now call a measurement of this magnetic field a “magnetotrichogram”, or MTG, where tricho denotes “related to hair”, in Greek.

**Figure 1:**
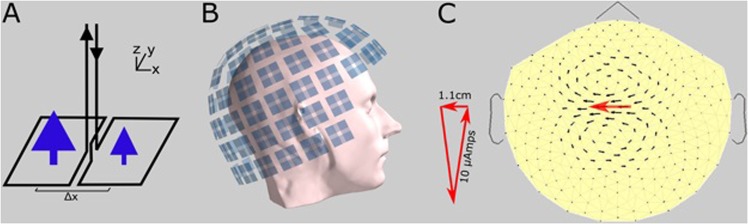
(**A**) Superconducting wire loops (pickup coil) of a single planar gradiometer. Blue arrows are the average normal magnetic field ${B}_{z}$ through each loop. The gradiometer output is proportional to Δ*B*_*z*_/Δx and is fed to one SQUID. The other half of a gradiometer pair is obtained by rotating the arrangement by 90°, not shown here, yielding the second output Δ*B*_*z*_/Δy, which is fed to a second SQUID. (**B**) The coil arrangement of our dcMEG inside the white liquid-helium dewar (the helmet in Fig. 2). There is a double gradiometer at 102 locations over the head. (**C**) An arrowmap in the format of a head, but not powered by a head but by a wire triangle instead, for calibration. This is a pure element of tangential (to the skull) current, a dipole, with a length of 1.1 cm, carrying 10 µA (microamp) dc, shown as a red triangle. It was placed against the white inner surface of the helmet. This source is shown on the arrowmap as a red arrow (of arbitrary length). The heavier black arrows, surrounding the red arrow, are seen to mimic the underlying current element. The arrangement of black arrows is the characteristic spatial response of our system to a pure current dipole. This measurement also calibrates the black-arrow lengths generally, in terms of the current element or “battery” underneath.

**Figure 2:**
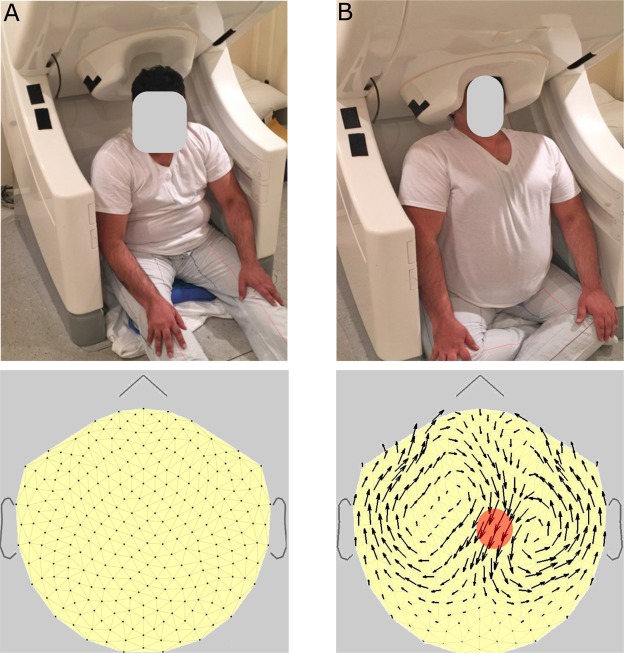
Arrangement for recording an MTG map, using the dcMEG detection and recording system, due to pressing a particular section of scalp. There are two steps. (A) Upper panel; Subject first sits slumped and relaxed, with his head outside the helmet, to get the “zero map.” Lower panel: Arrowmap, or MTG map, which has just been zeroed, by the operator. (B) Upper panel: Then the subject places his head inside the helmet, pressing the top of the head moderately against the inside top of the helmet. Lower panel: resulting MTG map, showing dc due to this head pressure. The red circle is the approximate area of pressure, and the black arrows inside the circle are the current generators (or “battery”) in the pressed follicles. These resistively generate the remainder of arrow-currents in the volume conductor of the scalp (more in Fig. 3 caption).

We here revisit and further study the MTG, but this time with an advanced detection and display system, the dcMEG. The detector in that early work consisted of two SQUIDs, fed by a pickup coil arrangement called a double planar gradiometer, which measured one location, shown partly in Fig. 1(A). However, with our present state-of-the-art dcMEG, we have 102 planar gradiometers (two SQUIDs each) at 102 locations, spaced in a helmet over the entire head (Fig. 2(B)). This is a large advance over the old one-location gradiometer. Further, the field in our new system is displayed as an on-line map of vectors (arrowmap), or the MTG map, where the arrows crudely mimic the flow of dc. Ideally, we wanted the arrows to perfectly mimic the currents in the head, so we would see the actual current flow. To do this, we have used a number of transformations. But we have attained that goal only very approximately. Using the actual element iΔl as a calibrating source at the helmet surface, we obtain the map shown in Fig. 1(C). All in all, an arrowmap comprising the MTG is a map of underlying dc currents, as is made plausible in Fig. 1(C) The way an MTG map is recorded as illustrated in Fig. 2.

To perform our study, standard sets of MTG maps were made of 10 male and 5 female subjects with normal hair, as well as one male and female with alopecia (non-functioning scalp hair follicles). A standard set consists of five MTG maps: 1. head in lightly, barely touching at the top; 2. head pressing above the left ear; 3. head pressing above the right ear; 4. head pressing at the top; 5. head pressing at the back. With three of the subjects, the natural tilt of the hair at various locations was compared with the MTG arrow angles at the pressure points. Various auxiliary measurements were carried out on some subjects, to gather further information. One was the measure of the MTG time-constant. On some subjects, we did multiple standard recordings to see repeatability, for a total of about 50 recording sessions. In these measurements, we also found two new sources of dc fields over the head: one source is magnetite particles in the brains of older subjects^8,9^ which could also be artifacts in the MTG map, if magnetized in the MRI, or in other ways. The other much smaller source of an unknown origin shaped like “wings” is also constantly present. The origin of these wings cannot be attributed to the heartbeat as the data segments use for Our goal is to quantify and better understand the dc from the follicles, using the dcMEG. However, generally, this work shows that the MTG opens up a new field entirely, in that it offers a unique tool for determining the vitality of hair follicles on the human scalp, and for studying hair diseases and baldness. One auxiliary use of this information is to see if this follicle signal can be subtracted out when looking at dc from the brain. That is, the follicle dc can be an unwanted artifact, in dcMEG brain studies. This is one long-range plan of the MEG community.

## Results

### Standard MTG maps

**Figure 3:**
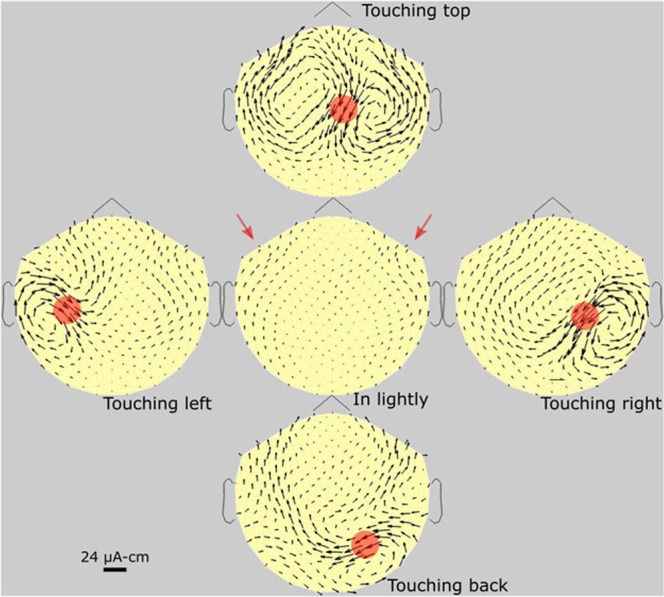
Subject #1. Standard set of MTGs of a 37 y/o full-headed male subject. The red circles are graphic additions, showing the approximate areas of pressure. The arrows under the circles are the dc sources or “batteries”, generating the resulting arrows in the resistive volume current of the scalp. The shapes of the arrow loops (currents) are determined by the variation of the shape and resistivity of the low-resistance scalp, as well as by the angle of follicle tilt. The two red arrows, also special additions, point to the non-follicle arrows we call “wings’, which are there without pressing. The calibration bar (24 µA-cm) refers only to the “battery” arrows under the red circles. It is the dipole strength of the generator; in this case, the generator arrow is about the length of the calibrating bar, or 24 µA-cm in maximum strength.

**Figure 4:**
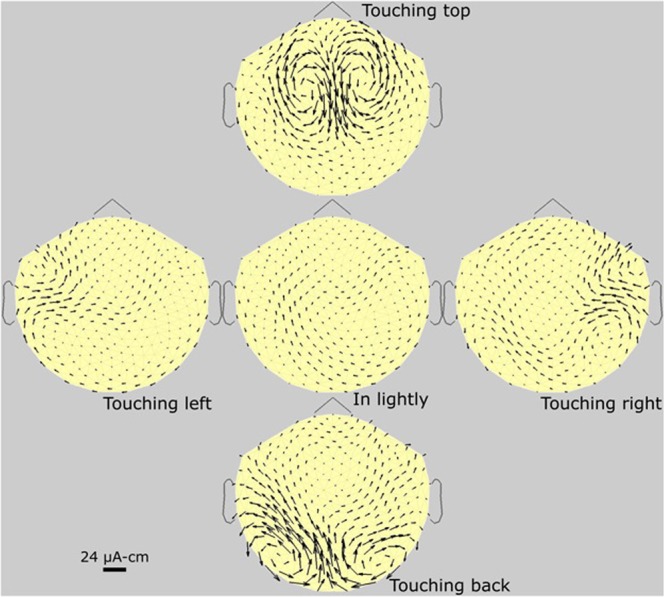
Subject #2. Standard set of MTG maps of a 25 y/o full-headed female subject. The arrows are somewhat smaller than those of Fig. 3, typical for females versus males. This could be due to a number of variables: less subject pressure, less follicle source strength, less follicle density, higher scalp resistivity, and other factors. Small wings are present.

**Figure 5:**
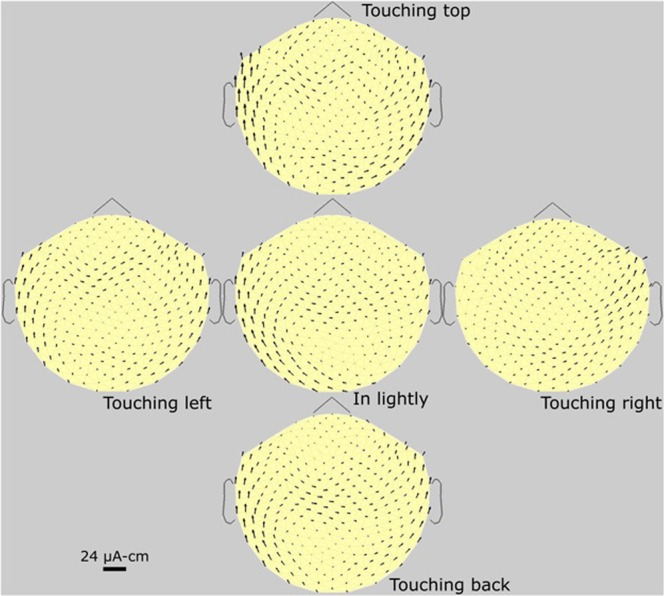
Subject #3. Standard set of MTG maps of a female subject with alopecia, hence with disabled scalp follicles. A standard set of another alopecia subject (male) is shown in SI, with many similar features. The four location signals are seen to be completely absent. However, the wings are seen to be present in all.

**Figure 6:**
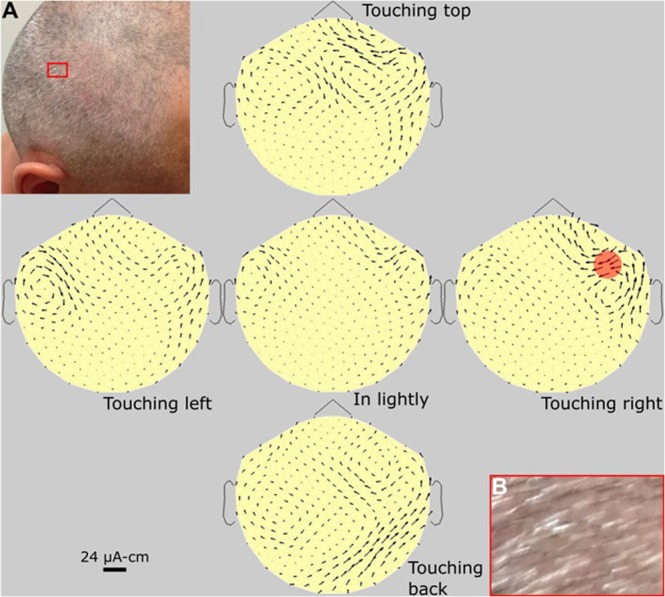
Subject #4. Standard set of MTG maps of a 38 y/o thin-haired male subject, with a recently shaved head, plus two photos. The arrows are significantly smaller than in Fig. 3, probably due to the less spatial density of follicles. The two photos are used to compare the actual hair-shaft (hence follicle) direction with the arrow-generator direction in the MTG map. A: photo of the head, B: Blow-up of the red box in A, showing directions of hair shafts.

Figures 3–6 are each a standard set of MTG maps, of four subjects. Standard sets of another three subjects are shown in supplementary information (SI). Figure 3 is of a male subject who usually has a full head of hair (no bald spots) although he occasionally shaved his head. this is the same subject as in the photo of Fig. 2. Therefore, this is as strong a set of arrows as we can expect. We here see “wings”, one of the two new phenomena. The maximum “battery strength” (touching back) is about 24 µA-cm. in agreement with the older result (Cohen *et al*., 1980. We note that the magnetic gradients from the follicles are larger than most reported DC levels from the brain (Boyer *et al*., 2012), in comparing these sources. Therefore, the MTG signals interfere with brain dc signals.

Figure 4 is a standard set of MPG maps of a full-haired female subject, showing somewhat smaller arrows than Fig. 3. Generally, in comparing all our full-headed female and male subjects (5 versus10), the female arrows are all about 10% smaller. Also, generally, arrow lengths are independent of hair color.

Figure 5 is a standard set MPG maps of a female with alopecia, where pressing the scalp gives no signal whatever, and the small remainder currents (“wings”) are of natural but unknown sources, located near or around the mouth. Figure 6 is that of another male subject, with less dense hair than in Fig. 3. In this figure, we show how the tilt angle of the follicles was investigated. We did this tilt study on three subjects with recently shaved heads, enough to convince us of this result: the direction of the arrows is always in line with the projection on the scalp of the hair shaft.

In comparing MTG maps, we found that they are all different; it is unlikely that any two subjects are identical. However, the pattern of each subject is reproducible, month after month. The two red arrows in Fig. 3 point to a new left-right arrow structure, which we call “wings,” not previously reported. This small-amplitude structure is present in about 80% of subjects, but as yet the exact origin is unknown. Figure 6 is a standard set MPG maps of a thin-haired male subject, where pressing the scalp gives significantly less dc magnetic field compared to the healthy subjects in Figs 3 and 4.

### The time-constant

**Figure 7:**
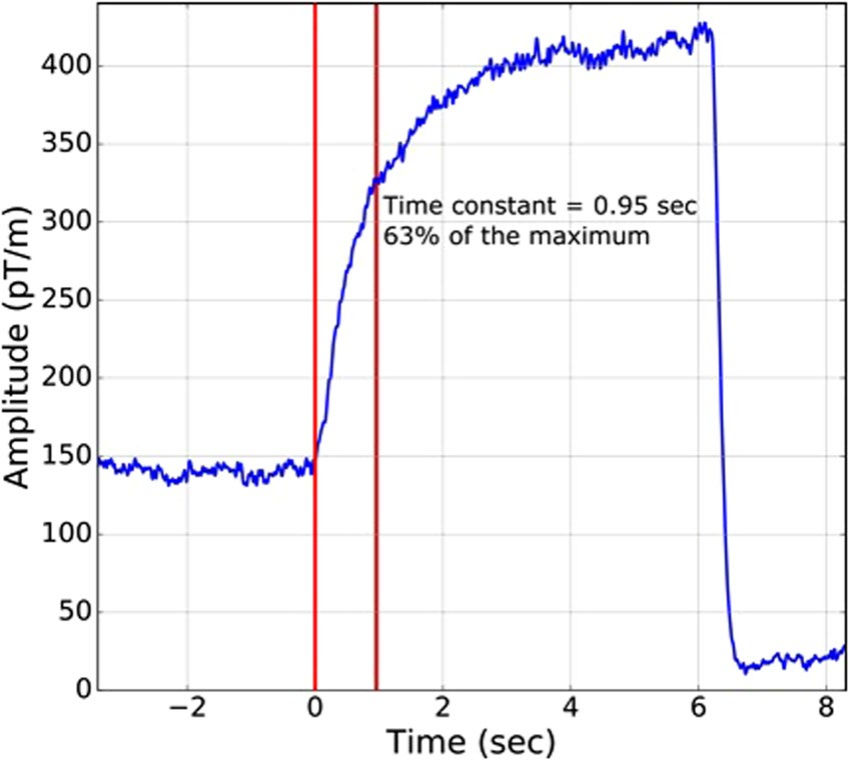
Rise-time measurement, of subject #1. The blue curve is the raw output of one gradiometer channel versus time. Before 0-time, the subject’s head is barely touching the top, too lightly for any follicle signal. At 0-time he rapidly presses his head upward and holds it steadily against the helmet top for 6 seconds, when he rapidly pulls his head out of the helmet. The second red line indicates 63% of the maximum rise, which is the time constant, by definition, seen here to be 0.95 seconds.

A time-constant measurement is shown in Fig. 7. This number appears to be about the same in other subjects, tested visually on-line. More operational details are given in Methods.

### Surface potentials

As explained in Methods, it was worthwhile to determine if we could see the follicle dc as a potential difference on the scalp surface. The experiment was performed on subject #1, because of his robust signal., and the fact that he shaved his head. We used one traditional EEG channel, with a traditional electrodes pair, pasted at various locations around the top of his head. Then we pressed the top point repeatedly. The electrodermal response was always seen to be greater than 20 mV, and after various trials and variations, we concluded that a valid measurement of the follicle potentials was almost impossible, or well beyond our technology.

### Comparing angles of tilt

The method of comparing the angle of the hair shaft leaving the skin, to the electrical generator angle of the follicle, is explained in Methods. This is partially demonstrated by the photos in Fig. 6. It was found, in the three male subjects measured, that the hair-shaft was always oppositely aligned (180 degrees) to the generator arrow, at a location just under the pressure point. We can generalize and suggest that this is always true. We here refer to the 2-dimensional projection of the angles onto the scalp surface.

### Using physical manipulations

The following simple physical manipulations to the scalp were attempted before the measurement, which could give information on the sources of the dc.

1. Application of hot and cold patches on the scalp.
2. Application of saline solution on the scalp.
3. Application of conducting paste on the scalp.
4. Reversal of vertical position of the subject by standing on his head.

All results were essentially negative, as the dc levels were changed less than ten percent by any of these manipulations.

## Discussion

### Follicles as the source

The above results contain two pieces of strong evidence that the MTG signals arise from the follicles:

First, there is the fact that the alopecia subjects show no pressing signals whatever, and alopecia subjects have disabled follicles; otherwise, the physiology of their skin is largely normal^10^. Hence this points strongly to the follicles, as the source. Second, the tilt of the hair leaving the scalp, on all subjects, is exactly opposite, at 180 degrees, to the generator arrows (under the red circles). This again points strongly as the follicles, as the source. Our results thus confirm the old brief report^3^ and extend our knowledge of this phenomenon.

We note that hair itself has nothing to do with our dc signal; it is only the follicles. The dc signal “does not know” about the hair length, cross-sectional shape, color, or oiliness, according to our measurements.

We believe the following is some of the simple physics Involved: Each follicle under the red circle becomes a battery when pressed, aligned in the hair-shaft direction. We consider the projections of the battery in the radial and tangential (to the skull) directions. The currents from the radial projection by symmetry, produce zero external magnetic field^2^, but the currents from the tangential component, flowing within the approximate spherical shell of the scalp volume conductor, do indeed produce an external dc field. The arrows in the MTG map roughly mimic these currents. Thus, we see the tangential currents flowing in the scalp, due to the pressing of the generators and the resistive result.

But what is the microscopic source, associated with a follicle? We suggest it could be the arector pili muscle structure, arranged circularly around the follicle, at least in tangential projection^11–13^ although there is a nerve structure around the follicle^13^. In the old study of dc fields over the body^3^, it was found that a major source of dc in the body was the change in the K+ concentration gradient over striated muscle fibers(not nerves). Pressing on arm and chest muscles changed the dc signal from those organs, and injection of K+ ions into the muscle gave enormous signals. therefore, in looking for local muscles, the only muscle associated with the follicle is the arector pili. Many microscopic fibers are a complicated source of many small dipoles, which look like a clean simple dipole, from “far away”, that is, at the gradiometer files.

To avoid complexities from alopecia progression, we selected two subjects with complete total alopecia. This is a possible limitation of this study and therefore, in future work, we will be studying subjects at different stages of alopecia.

Concerning sex differences of MTGs, we had expected some differences and designed our study accordingly. It was surprising, therefore, to see only the minor difference of somewhat smaller arrows in females.

### No surface potentials

Some organs give both measurable surface potentials and magnetic fields, such as the heart and brain, but that is In the ac domain. In contrast, in the dc domain, the internal organs, such as hair follicles give only the magnetic field, such as the MTG signal, therefore it is unique, hence a potentially valuable technology. As far as we can tell, there is no other non-invasive way of measuring the electrical activity of the follicles. The same dc magnetic uniqueness may eventually apply to other internal organs, as well.

### Final remarks

There is a rich world of direct current (dc) in the human body, produced by slow chemical reactions generally, as yet scarcely studied and understood. Most internal dc events are usually not detectable by surface (skin) potential measurements, because of large interfering skin potentials, which mask the smaller potentials from internal dc. However, the weak external dc magnetic fields generated by the internal dc are detectable by the SQUID, which therefore offer a window into this new world. There is “the cold water effect,” for example^14^. This is one out of perhaps many reflexes. In our case, using our dcMEG, we see the magnetic fields from the follicles. Thus, this appears to be a new unique way to study hair diseases and baldness. Further, our follicle work also shows a barrier which must often be overcome in measuring the deeper dcMEG from the brain, an eventual important goal.

## Methods

### The subjects

The participants were 15 normal subjects (5 females), and 2 with alopecia (non-functioning follicles), in the age range of 19 to 79 y/o. d. Informed consent approved by the institutional review board (IRB, Mass. General Hospital, Boston, MA) was obtained from all participants. All methods were carried out in accordance with relevant guidelines and regulations. All participants completed the written informed consent before the experiment and received monetary compensation for their participation.

The normal subjects were healthy, by which we mean that they presented with no obvious health, especially dermatology problems. Also, they were questioned at length concerning magnetic artifacts and presented with no obvious ferromagnetic material in their mouths (dental) or body, or with any recent MRI, because this would have magnetized not only dental or body ferromagnetic material, but also magnetite particles in the older brains^9^. Only the imaging of MEG was required from these subjects, as was approved by the IRB. It was not necessary to perform CT or MRI scans to look for metallic (ferromagnetic) artifacts in the head, because the dcMEG is much more sensitive to the ferromagnetic material, in this regard, and would easily respond to artifacts not seen in a CT or MRI scan. We were fortunate in that three of the male subjects shaved their heads before or during their measurement cycle, including subjects #1 and #4, whose results and photos are illustrated here.

### The dcMEG

The detector in the early work^3^ consisted of only one pair of pickup coils called a planar gradiometer pair. It was located in a small tail of a large dewar, and any part of the subject’s body could be placed at the tail, but only at one location at a time; therefore, a dc mapping over the whole head, for example, was most cumbersome, because it involved a long sequence of placements. But with our present state-of-the-art MEG system we now have many planar gradiometer pairs spaced over the entire head, and one MTG scan measures the entire head.

Our MEG is manufactured by the Elekta Co. and is the model called VectorView. Later models have the same pickup coils and are therefore suitable as well. These models contain 306 SQUID (Superconducting Quantum Interference Device) detectors, arranged in groups of three, at 102 locations over the head, where each SQUID is fed by a pickup coil sensing the ambient field. There are therefore three pickup coils at each of the 102 locations. One coil is a magnetometer (simple loop) and the other two coils make up a planar gradiometer pair. We use only the gradiometers, that is, 204 SQUIDs, not the 102 magnetometers.

To use our modern Elekta VectorView MEG system for dc, we not only lowered the bandwidth down to dc, but also modified the 102 planar gradiometer pair outputs, passing them through various transformations, onto a resulting online map of arrows, as in the lower panels of the Fig. 2. We chose the gradiometer-based system over a magnetometer-based system because of the fluctuating dc field background. The gradiometer-based system is normally immune to this fluctuation, especially in a heavily shielded room^15^, like the one used for this study.

In preparation for the dcMEG, the subject changes into non-magnetic clothes, and his scalp and/or hair is washed, to remove any artefactual ferromagnetic particles, common in the dust of an urban environment. The position and orientation of the head with respect to the MEG helmet were recorded with the help of four head position indicator (HPI) coils^16^.

To avoid possible contamination by the heartbeat, data segments between the two heartbeat are chosen for the subsequent analysis.

### Setting up the arrows for the dcMEG

We set up the computational machinery by starting with the arrowmap. We note that the original gradiometers take the gradient in a spherical coordinate system, i.e. along latitude and longitude. Therefore, they are mapped to the virtual gradiometers taking the gradient in the Cartesian coordinate system. The Hosaka–Cohen transformation^17^ is then applied to these gradiometers:

$$\overrightarrow{{v}_{n}}=\frac{\partial {B}_{z}}{\partial y}{\hat{x}}_{n}-\frac{\partial {B}_{z}}{\partial x}{\hat{y}}_{n}$$

Where $\overrightarrow{{v}_{n}}$ are the transformed gradients (Hosaka–Cohen transformation) at each location, ${B}_{z}$ is the z component of the magnetic field, $\frac{\partial {B}_{z}}{\partial y}$ and $\frac{\partial {B}_{z}}{\partial x}$ are the output of the virtual planar gradiometer, ${\hat{x}}_{n}\,$and ${\hat{y}}_{n}$ are the unit orientation vectors.

It can be shown that each arrow has the units $k\mu A/c{m}^{2}$, where *k* is a constant of proportionality and *μA* is microampere. Each arrow is therefore proportional to the local current density. This applies to arrows located over a resistive medium. However, there is another description as well. When there is a generator below the measuring point, the overlying arrows are a mimic of the dipole strength of the generator. We choose the calibration in Fig. 1(c) thusly: We let the largest arrow (instead of the total tight group) reflect the underlying dipole, thus giving us the calibration bar at the lower left of each MTG map. The bar refers only to tangential generators, essentially a tangential battery, driven by pressure on the scalp. We don’t see the radial component, because of symmetry, a well-known phenomenon in the acMEG literature^2^.

### Measuring the time-constant

Knowledge of the time-constant of the follicle signal is certainly necessary for eventually understanding the source of this follicle phenomenon. We chose a simple and direct method to obtain this information. We measured the turn-on curve of the magnetic field, due to a sudden step-on pressure application. We chose the raw signal from one of the SQUIDs, which had a large robust response to the pressure turn-on. The most effective location was thought to be the “touch-top” area, in a subject who had a full head of hair with a robust touch-top signal, in this case subject #1. He first put his head in lightly, then after the particular signal was steady, he was instructed to “press-up”. He made contact with the helmet in a time short compared to the signal build-up, yielding a good signal curve, as shown in Fig. 7.

### Measuring the surface potentials

It is important to see if the MTG has a potential counterpart, that is, an ETG on the scalp surface; is the MTG unique, or is there another way to see the electrical activity of the follicles? The equivalent dipole generator (radial as well as tangential) must certainly produce a dc potential on the scalp surface. The question is: Can it be measured? This doubt is due to the large electrodermal skin potential of 30 mV^18–20^ which has variations which can mask the follicle signal. Assuming a radial source dipole strength of 30 uA-cm, located at 3 mm below the surface of the scalp, we have calculated a maximum potential difference between two nearby surface points of about 4 mV. It may thus be barely possible to see this, through the range of electrodermal variations, say in the range of 5–20 mV. Therefore we did attempt a measurement of subject #1, who had shaved his head at one point in time. As shown in Fig. S4, EEG paste was put on the scalp measuring points, then EEG electrodes were embedded into the paste (normal EEG pasting). After this preparation, the follicle signal was activated by a simple finger pressing on the scalp by the experimenter; there was no need to use the dcMEG.

### Inverse solution

The position and area of the head surface pressed against the helmet were estimated from the gradiometer raw data in the following way. For the forward solution, the head model: T1-weighted, high-resolution MPRAGE (Magnetization Prepared Rapid Gradient Echo) structural images were acquired on a 1.5 T Siemens whole-body MRI (magnetic resonance) scanner (Siemens Medical Systems) using a 12- channel head coil.

The structural data of the head was preprocessed using FreeSurfer^21^. To compute the forward solution; the skull was segmented using the watershed algorithm in FreeSurfer. Head surface was then discretized in three-dimensional source space. Source space consisted of a grid of 1568 dipoles.

The current dipole distribution was estimated using the distributed solution approach employing minimum-norm estimate (MNE) with free orientations. MNE estimates the sources as the solution to a linear imaging problem and estimates source density image jointly for all the dipoles that best fits the data and favors solutions that are of minimum energy (or L2 norm). Unlike parametric methods like single or multiple dipole fit which assumes sources can be represented by a few equivalent dipoles, distributed solution estimate source density image jointly for all the dipoles. The regularized (regularization = 0.1) noise covariance matrix used to calculate the inverse operator was generated using empty shielded-room data. The dynamical statistical parametric mapping (dSPM)^22^ map was calculated by dividing MNE value with the projection of the estimated noise covariance matrix at each source point (Fig. S6).

### Comparing angles

Hair, as it leaves the skin, has a characteristic tilt (angle), in both man and animals^23–25^. The old report^3^ noted a connection between the hair tilt angle and the angle of the magnetic gradient, and we here more fully investigate this connection. On three male subjects with shaved heads, we photographed the scalps and plotted the tilts projected onto the head, usually on a foam head. These tilts were then compared with the measured arrows under the pressure points, that is, the generator angles. An example of part of this process is shown in Fig. 6, on the scalp of subject #4.

## Supplementary information

Supplementary Information

## Data Availability

All data will be made available upon request.
