# Supplementary material for: Magnetotrichography: Measuring the dc magnetic field produced by hair follicles
Source: Sci Rep. 2019 Oct 30;9:15624. doi: 10.1038/s41598-019-52110-y (PMC6821812; doi:10.1038/s41598-019-52110-y)
Supplement: Supplementary file 1 — Supplementary Information [file 41598_2019_52110_MOESM1_ESM.pdf]

SUPPLEMENTARY INFORMATION

Magnetotrichography: Measuring the dc magnetic field produced by hair follicles

Sheraz Khan[1, 2] and David Cohen[1,2,3 *]

[1]Radiology, Massachusetts General Hospital, Harvard Medical School, Boston, MA, USA
[2]Athinoula A. Martinos Center for Biomedical Imaging, Boston, MA, USA
[3]Francis Bitter Magnet Lab, Massachusetts Institute of Technology, Cambridge, MA, USA

***Corresponding author:**
David Cohen, Ph.D.
Athinoula A. Martinos Center for Biomedical Imaging
Massachusetts General Hospital
149 13th Street
Boston, MA-02129, USA
Phone : +1 617-547-2658
Fax : +1 617-948-5966
E-mail: davcohen@mit.edu
http://davidcohen.mit.edu

More standard sets of MTGs.

We here show the standard sets of three more subjects.

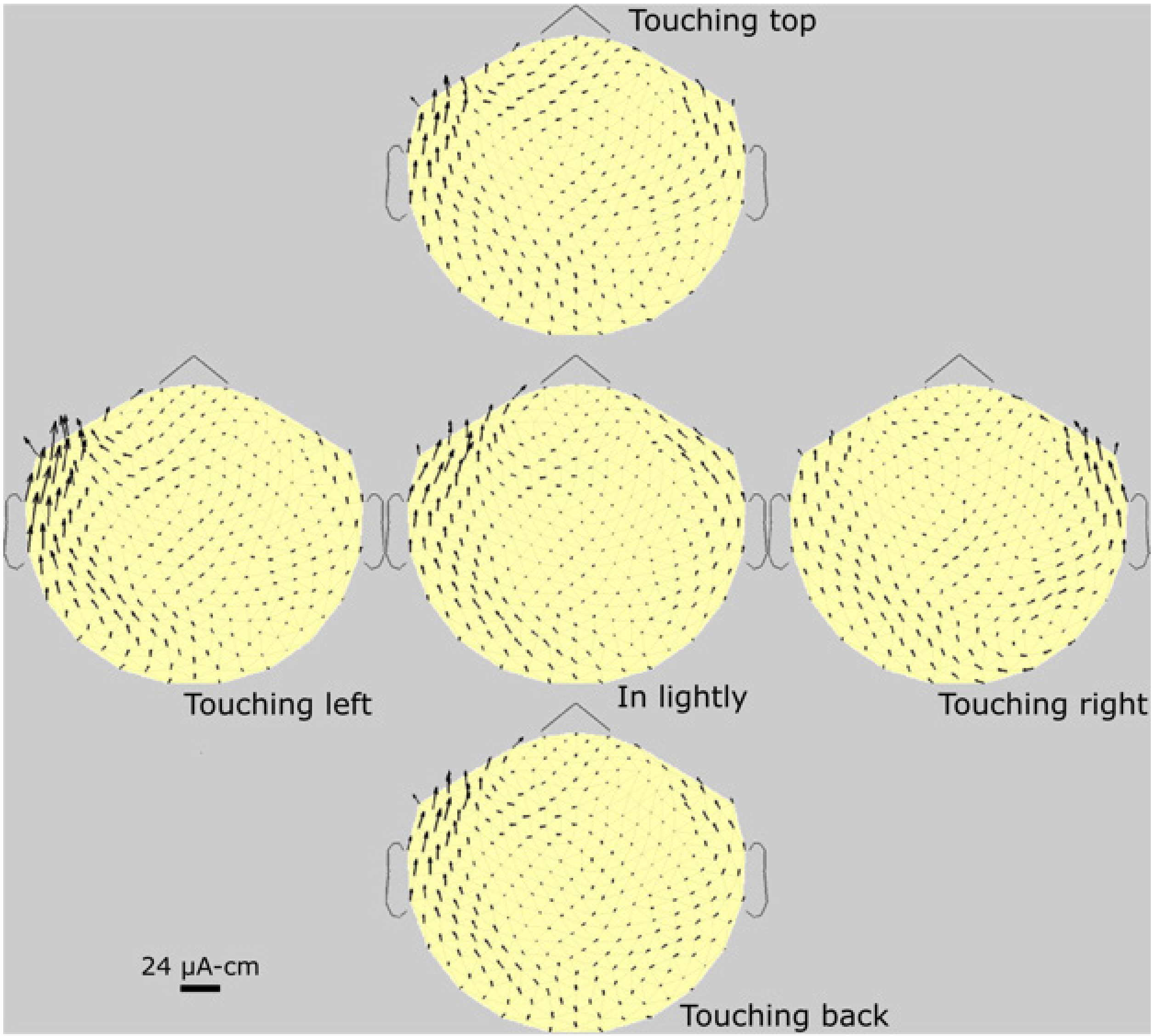

Fig. S1. Subject #5. Standard set of MTG maps of the second alopecia subject, a 32 y/o male. The source of the arrows at the far left, when pressing left, is unknown. It is unlikely they are follicle signals. They are most unusual. In general, elsewhere, there are no follicle signals, as expected. There are, however, prominent wings, on all.

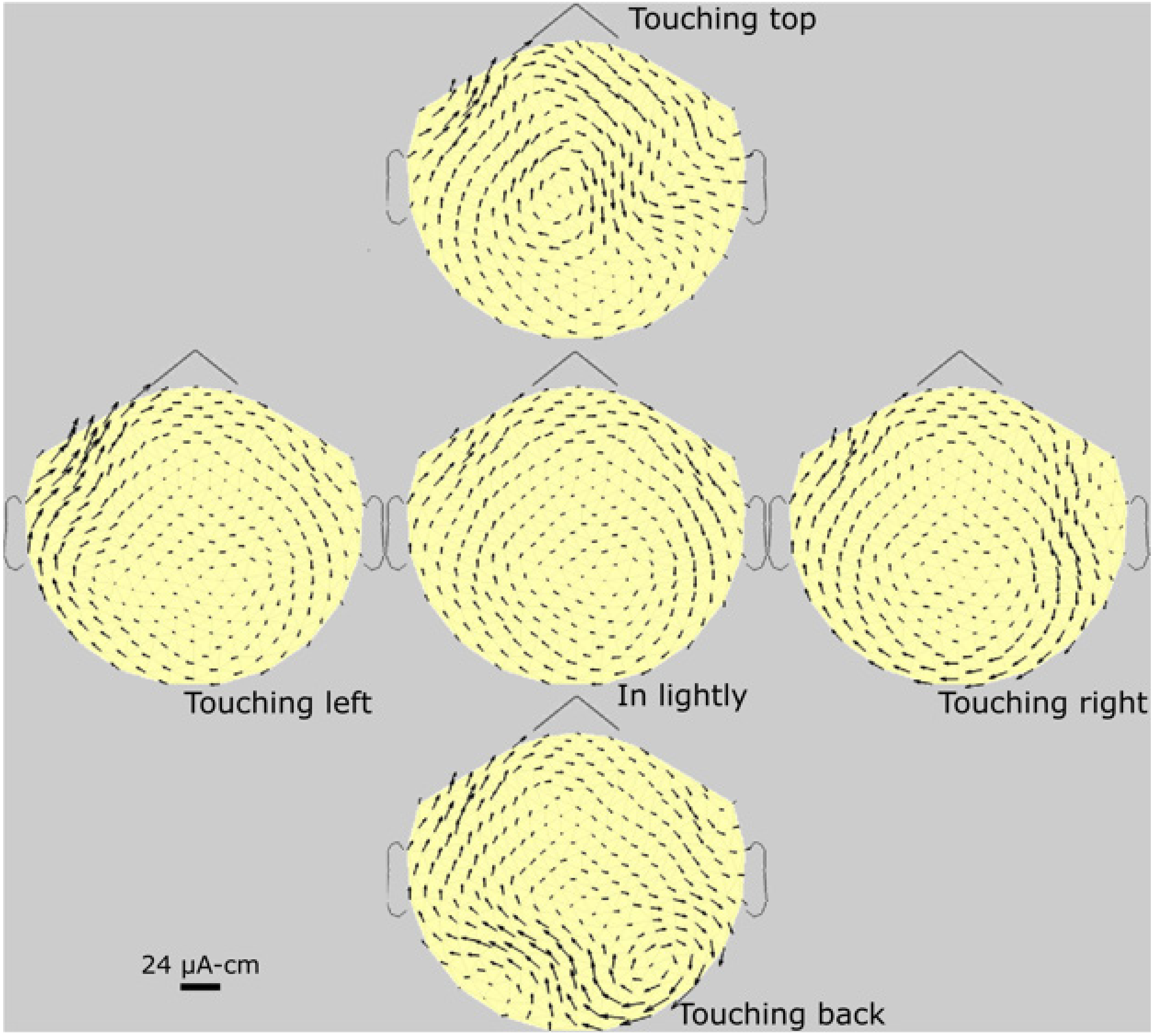

Fig. S2 Subject #6: Standard set of MTG maps of a 50 y/o full-headed female, with a large head. These maps are not typical. The center arrowmap (head in lightly) indicates some light touching, both left and right, due to her head size. In her case, there is probably no clear press-left and press-right stimuli. This is not a clear, good standard set.

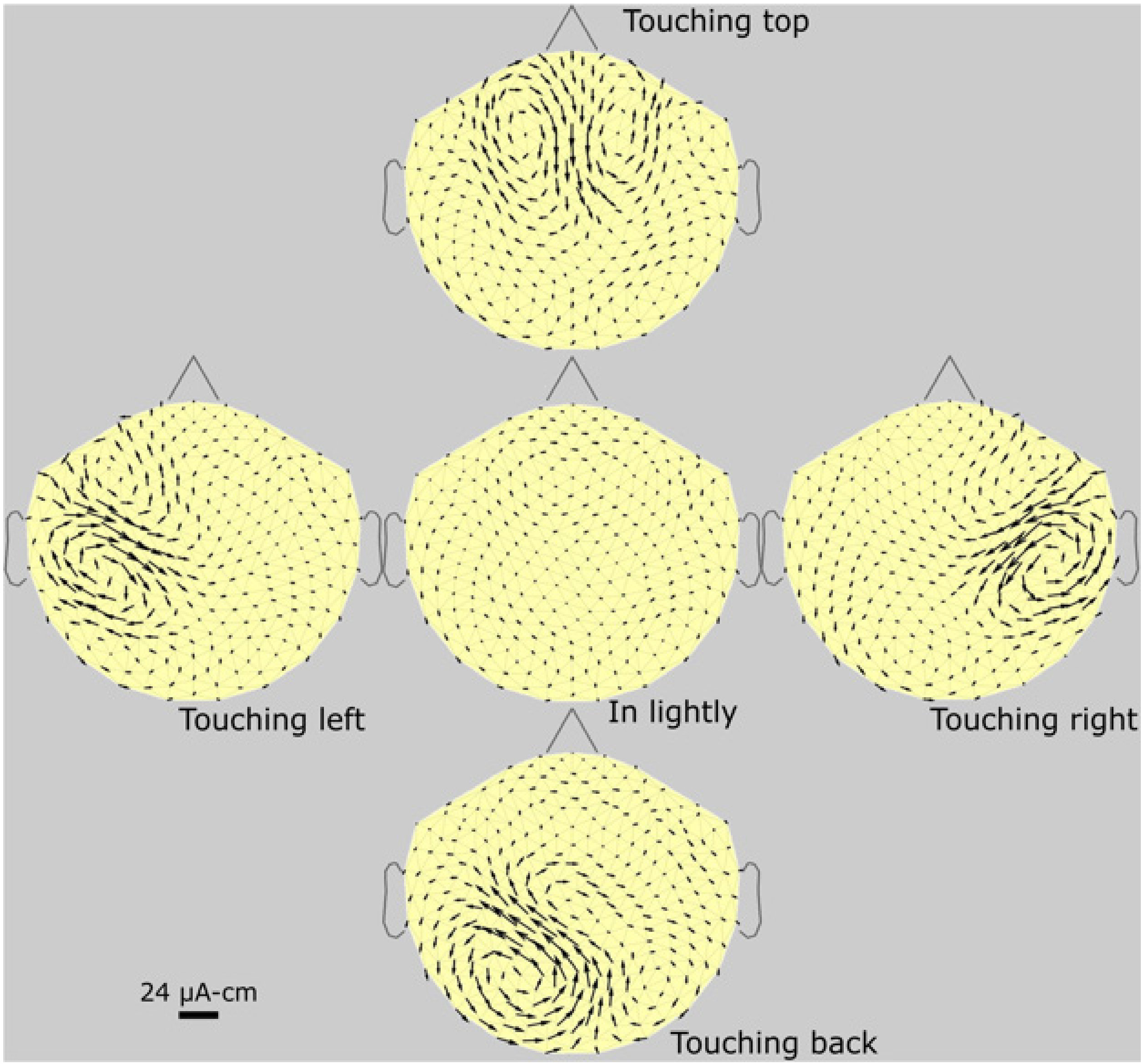

Fig. S3, Subject #7. Standard set of MTG maps of an average-haired 25 y/o male. Notice there are no wings in his case. Could this be related to the fact that this subject is unusually thin?

Photos of the scalp of subject #1

We were fortunate in that three of the male subjects had their heads shaved before or during their repeated measurements, often over a period of months. Photos such as these were used to document the hair-shaft tilts as they leave the scalp, in comparison to the arrow tilts.

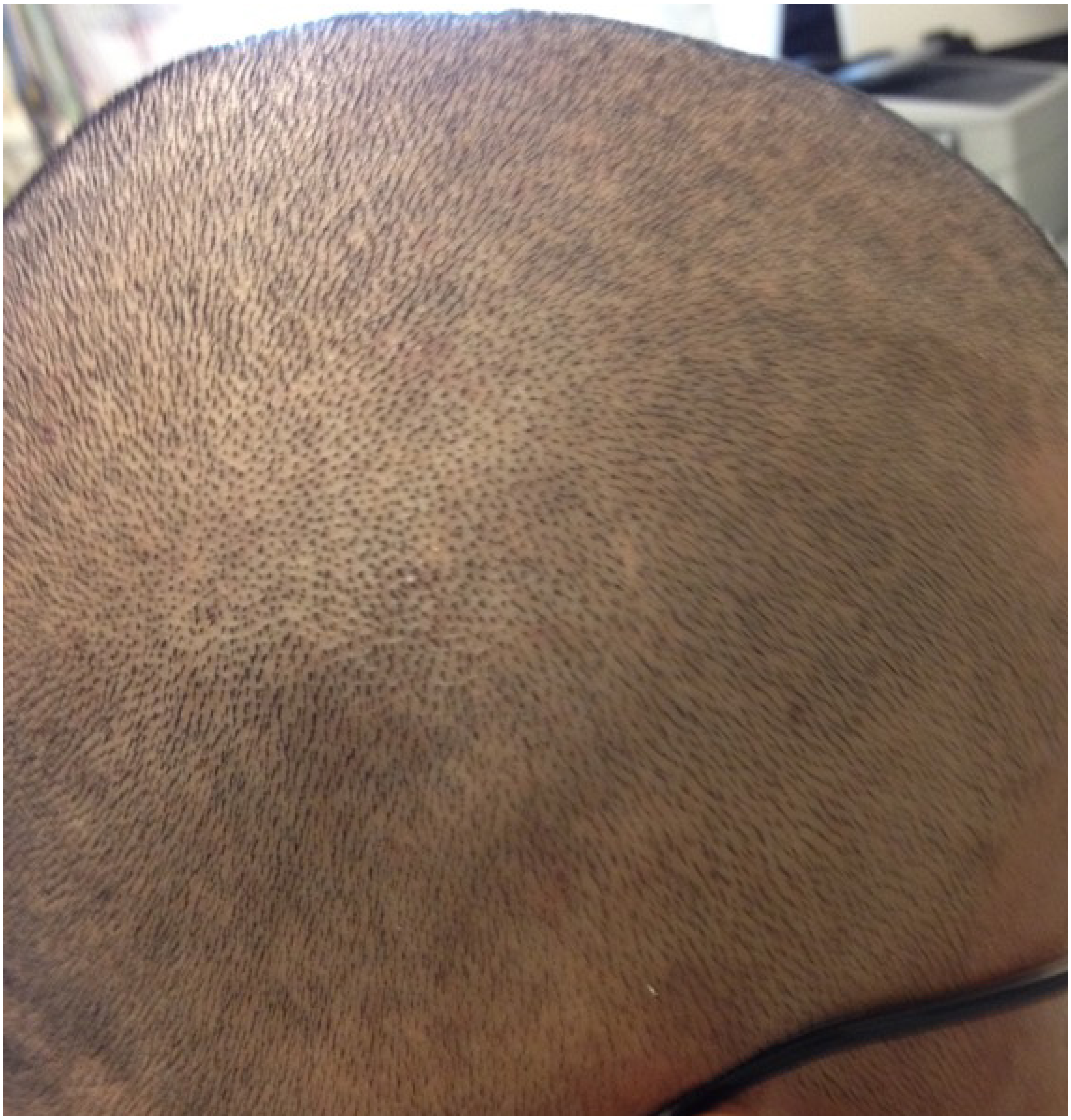

Fig S4. Recently shaved head of subject #1. Notice how easy it is see the hair-shaft directions.

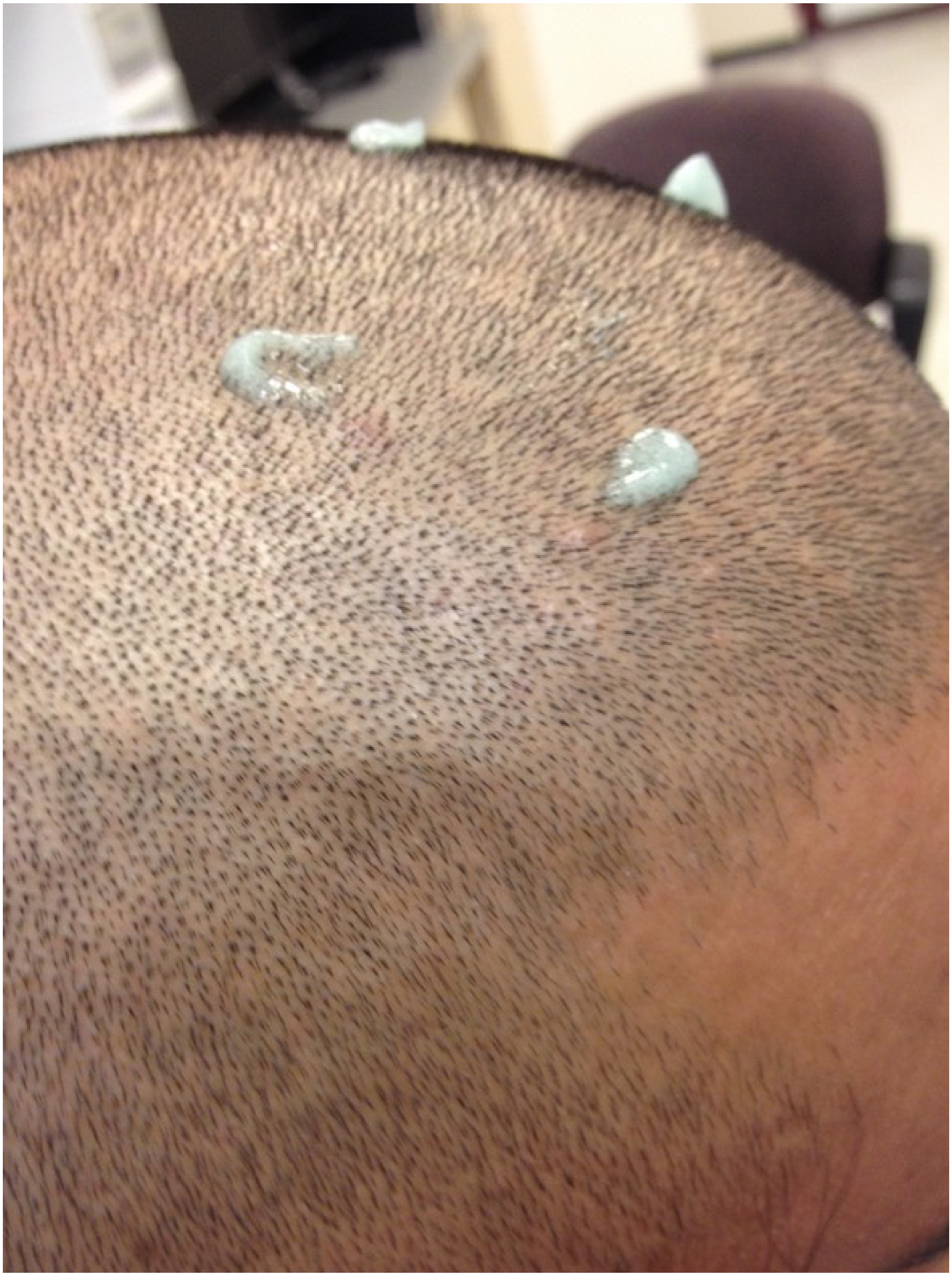

Fig S5. Preparing to measure surface potentials on subject #1, with EEG paste. The EEG electrodes will then be embedded into the paste, The result is that no clear potentials due to follicles are seen because they are masked by electrodermal potentials.

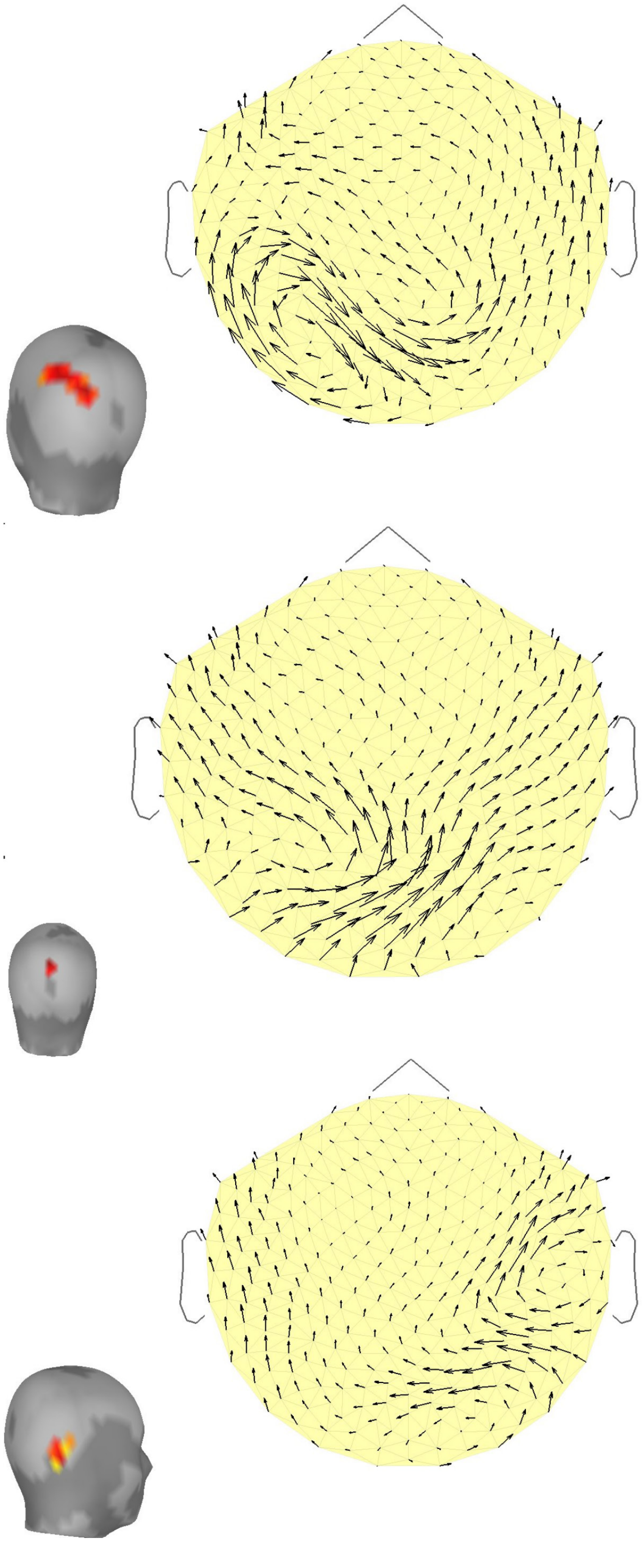

Fig S6. Location of the scalp area pressed as estimated by the minimum norm inverse solution.
